# Patient and carer perceived barriers to early presentation and diagnosis of lung cancer: a systematic review

**DOI:** 10.1186/s12885-018-5169-9

**Published:** 2019-01-08

**Authors:** Shemana Cassim, Lynne Chepulis, Rawiri Keenan, Jacquie Kidd, Melissa Firth, Ross Lawrenson

**Affiliations:** 1 Waikato Medical Research Centre, University of Waikato, Hamilton, 3240 New Zealand; 2 School of Nursing, University of Auckland, Auckland, 1023 New Zealand; 3 Waikato Medical Research Centre, Waikato DHB Campus, Waikato Hospital, Hamilton, 3240 New Zealand

**Keywords:** Barrier to diagnosis, Early presentation, Lung cancer, Cancer care, Primary care, Delay to diagnosis

## Abstract

**Background:**

Lung cancer is typically diagnosed at a late stage. Early presentation and detection of lung cancer symptoms is critical to improving survival but can be clinically complicated and as yet a robust screening method for diagnosis is not available in routine practice. Accordingly, the barriers to help-seeking behaviour and diagnosis need to be considered. This review aimed to document the barriers to early presentation and diagnosis of lung cancer, based on patient and carer perspectives.

**Methods:**

A systematic review of databases was performed for original, English language articles discussing qualitative research on patient perceived barriers to early presentation and diagnosis of lung cancer. Three major databases were searched: Scopus, PubMed and EBSCOhost. References cited in the selected studies were searched for further relevant articles.

**Results:**

Fourteen studies met inclusion criteria for review. Barriers were grouped into three categories: healthcare provider and system factors, patient factors and disease factors.

**Conclusions:**

Studies showed that the most frequently reported barriers to early presentation and diagnosis of lung cancer reported by patients and carers related to poor relationships between GPs and patients, a lack of access to services and care for patients, and a lack of awareness of lung cancer symptoms and treatment. Addressing these barriers offers opportunities by which rates of early diagnosis of lung cancer may be improved.

## Background

Lung cancer is one of the most common causes of death from cancer worldwide [1]. It has been estimated that nearly one in five deaths globally are due to lung cancer, with 1.59 million deaths reported in 2012 (19.4% of the total). Overall survival rates for lung cancer are poor, with five year survival rates being 10–20% post diagnosis in most countries including New Zealand, Canada, Australia and Sweden [2–4]. A key reason for poor outcomes in lung cancer survival is the fact that it is typically diagnosed at a late stage when the patient has presented with symptoms. Population based screening for early stage lung cancer using LDCT (low dose computerised tomography) has been shown to be effective in identifying cases at an earlier stage and in reducing lung cancer mortality [5]. However, there is a high cost and a high false positive rate in using LDCT as a screening test [6]. Consequently uptake has been very slow and further research is ongoing in assessing whether there are particular high risk populations where screening for lung cancer can be justified.

An alternate strategy is to focus on the reason for late diagnosis. These can be due to patient factors, system factors and tumour factors [7]. Lung cancer symptoms can be different from person to person, and while most people show at least some early symptoms, some show none [8, 9]. Moreover, symptoms particular to lung cancer may be subtle and not directly related to the lungs and chest (e.g. tiredness and weight loss are sometimes the presenting symptom) [8, 9]. Consequently, symptoms are often misinterpreted or misattributed by both patients and General Practitioners (GPs). Misinterpretation can be exacerbated by the existence of co-morbidities, which can result in delayed diagnosis or referral [10–14]. Cross-cultural variations across nine countries have shown differences in the delay in reporting symptoms, ranging from 7 days to 6 months [15]. Early recognition of lung cancer symptoms combined with early medical help–seeking behaviour can have the potential to increase survival and decrease mortality from lung cancer [11, 16–18]. However, the proportion of patients who are identified with early stage cancer and receive curative surgery is low, with studies showing a prevalence of between 15 and 20% [19–22].

In saying this, recent research also points out that, although shortening of diagnostic intervals can result in clinical benefits for some patient groups (e.g. in terms of diagnosis and post-diagnosis cancer management in primary care), for others, it may not necessarily translate to improved outcomes. This can be due to various broader reasons including the symptom signature of lung cancer [9] or the patients’ perception of their experience within the healthcare system [23].

Overall, to maximise patient survival from lung cancer, early detection remains an imperative factor, alongside prompt referral. It is therefore necessary to increase the proportion of patients diagnosed with early stage disease. However, numerous studies indicate that there are significant barriers towards help-seeking behaviour and diagnosis. The objective of this systematic review was to explore and document the barriers to early presentation and diagnosis of lung cancer, identified by patients and carers (including those specific to indigenous and ethnic minority groups).

## Methods

### Search strategy and selection criteria

**Fig. 1:**
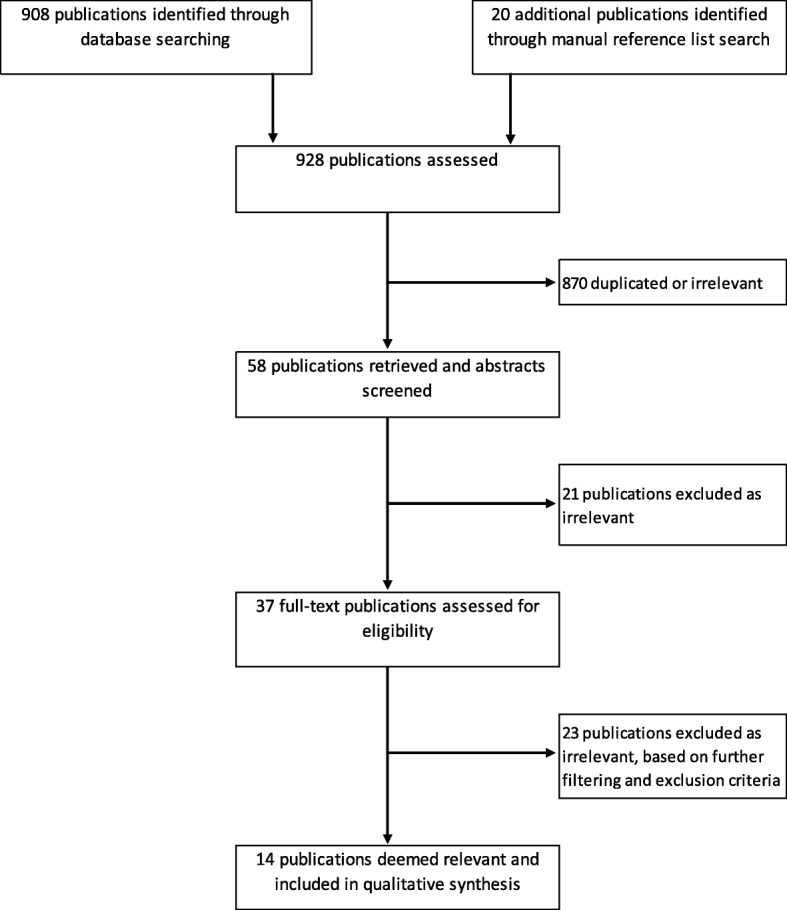
Process of literature selection for barriers to lung cancer diagnosis flow chart

Three major databases, Scopus (1960–2017), PubMed (1945–2017) and EBSCOhost (1888–2017), were searched from 23rd November to 8th December 2017, for papers published in English prior to December, 2017. Text words or keywords used in the search were “lung cancer” and “barrier”, “obstacle”, “difficult* (difficulty)”, “problem”, or “diagnos* (diagnosis/diagnostic)” combined with (i.e., AND) “general practi* (general practice/ practitioner)”, “primary care”, or “family practice”. Inclusion criteria for the extraction of articles from the databases were original, qualitative studies, published in peer reviewed journals, and a focus on patient and family or carer perceptions of barriers to early presentation and diagnosis of lung cancer. Accordingly, our exclusion criteria were literature reviews, quantitative analyses, studies focusing solely on prevention (e.g. screening) and a focus only on GP or health care provider perceptions of barriers to lung cancer diagnosis. It should be noted here, that our focus on only qualitative analyses was to identify key themes relating to patient perceived barriers to lung cancer diagnosis. By including quantitative studies in our review, we would have risked being in danger of leaving out important themes voiced by patients themselves, as barriers identified based on quantitative surveys or questionnaires tend to be predetermined. Furthermore, our search did not extend to non-English language studies or grey literature. References cited in the selected studies and any literature reviews with broadly similar search criteria were searched for further relevant articles. Figure 1 shows a flow chart of the process of selecting and including relevant studies for this review according to the PRISMA guidelines [24].

The inclusion of articles published only in peer reviewed journals was our first method of assessing the quality each study reviewed. The quality of each study was also assessed using the CASP checklist for qualitative research [25]. All studies yielded generally strong scores in the domains of validity, results and local relevance or value.

### Categorisation of perceived barriers

Walter et al’s [7] model for examining pathways to cancer diagnosis was used as a guide for identifying and grouping barriers to diagnosis in the studies selected. We particularly focused on the “contributing factors” section of the model, which indicates that healthcare provider and system factors, patient factors and disease factors contribute to delays in cancer diagnosis and initiation of treatment. It should be noted, however, that while co-morbidities are listed under patient factors in Walter et al’s [7] model, we included them as disease factors in our review due to the nature of symptom presentation particular to lung cancer, as discussed previously.

### Data extraction

The selected articles were reviewed and the following data were extracted and compiled into a table: general information about the article (authors’ names, year of publication, and methodology); study location; participant information (participant group, ethnicity, and number of participants); and a brief description of the findings, specifically the barriers to early presentation and diagnosis of lung cancer relating to health care system, patient and disease factors. A number of studies that had multiple participant groups (i.e. patients, family members, GPs and other service providers), explored multiple types of cancer (i.e. lung, prostate, breast and colorectal), used mixed methods (both qualitative and quantitative), and had a primary focus that went beyond identifying barriers to early presentation and diagnosis (e.g. developing an intervention) were included, but noted accordingly.

## Results

We identified 908 publications through our database search, and an additional 20 from a manual reference list search. By screening article titles, 870 were deemed to be duplicates or irrelevant based on topic. The abstracts of the remaining 58 articles were screened, from which 21 were excluded as they examined other cancers, not including lung cancer. Full articles were assessed on the remaining 37 potential publications. Of these, 23 were excluded as they focused on lung cancer screening, they did not examine patient and carer perspectives of barriers to diagnosis and/or they were literature reviews or quantitative analyses. The remaining 14 articles were included in our review. The characteristics and results of the studies are summarised in Table 1.

**Table 1:** Characteristics and results of studies relating to barriers to lung cancer diagnosis

Study	Study design				Findings (Barriers)			
	Methodology & sampling	Country	Ethnicity	Participant group	Number of participants	Healthcare provider and system factors	Patient factors	Disease factors
Sharf, Stelljes & Gordon (2005) [35]	M: qualitative (interviews) S: Hospital - participants recruited through pulmonary conference for ongoing cohort study, and review of pathology reports	United States	Not reported	Patients	9	Inadequate information provided to patients; i.e. regarding what to expect in relation to the disease and treatment; Lack of established relationship between physician and patient; discontinuity of care; long waiting times.	Patients took action to improve their suspected lung problems or health in general on their own, as an alternative to physician’s recommendations; Patients minimizing risk factors, possibility of a cancer diagnosis, or severity of illness. Patient relies on positive experiences with illness or treatment experienced by self or others; Fatalism and faith: patient’s emphasis on the importance of powers that outweigh self-control, i.e. fate or God; Patient’s distrust and suspicion of health information, medical procedures, motives of doctors or other health authorities. Patient focuses on negative experiences and expectations, past, present and future; Patient’s capacity or desire to live without knowing their diagnosis; Patient’s denial or questioning of the utility of treatment or procedure; Patient postponing treatment or delays seeking medical treatment after self-recognition of symptoms; Patient wanting to avoid pain or discomfort caused by medical procedures; Patient prioritizing his/her remaining quality of life; patient not being able to afford private insurance, thus limiting options for seeing specialists and obtaining medications.	
Tod et al. (2008) [11]	M: qualitative (interviews) S: community & hospital - respiratory physician and lung cancer nurse specialists	United Kingdom	Not reported	Patients and survivors	20 (18 patients, 2 survivors)	Media messages interacting with cultural tendencies to reinforce the belief that people should not use primary care services unless a problem was extreme.	Tendency to attribute symptoms to other acute and chronic conditions; Patient’s poor knowledge and awareness of lung cancer risks, symptoms and treatments; fatalistic beliefs and fear of death and cancer diagnosis (due to lack of awareness of lung cancer treatments); Patient’s fear of a medical consultation and being seen as a timewaster further prompted delay; previous bad experiences; Non- or ex-smokers delayed in reporting symptoms because of an expectation, based on previous experience, that they would be stigmatized as a smoker and blamed for their illness; patient placing great value on stoicism, not complaining and “putting on a brave face”; Lifelong patterns of poor healthcare utilization resulted in patient’s reluctance to see a GP unless symptoms were severe.	Symptom experience: wide variation in symptoms and therefore lack of a clear symptom profile.
S. E. Hall et al. (2008)^a^ [29]	M: qualitative (interviews) S: cancer notifications in Western Australia Cancer Registry	Australia	Not reported	Patients and GPs	41 (14 patients)	Quality of care: quality of communication from and between care providers and communication skills in general.	Misinterpretation or minimization of symptoms; financial aspects of cancer care (rural patients); transport costs and time.	
Smith et al. (2012)^a,b^ [26]	M: qualitative (focus groups) S: General Practice and ‘Breathe Easy’ group - specialist lung cancer nurses	Scotland	Not reported	Patients, families, GPs and other service providers (psychologists, sociologists, respiratory physician, health services researcher)	7 patients (plus spouses/partners)	Difficulty in making appointments due to lack of advice on making appointments and exactly what to ask when contacting GP; lack of involvement of the entire GP team in the process (e.g. receptionist as first point of contact, triage by practice nurses, GPs)		
Emery et al. (2013)^c^ [30]	M: mixed methods (medical records & interviews) S: rural cancer nurse coordinator, Cancer Registry and treating clinician	Australia	Not reported	Patients	66 (8 lung cancer patients)	Distance to health care	Misinterpretation or minimization of symptoms; low risk perception; stoicism (particularly of men)	Symptom experience
Walton et al. (2013) [36]	M: qualitative (interviews and focus groups) S: Hospital -primary physician and hospital physician	New Zealand	New Zealand European, Māori	Patients and family members	39	Access to services - patient getting delayed in the system (hospital booking systems, difficulties faced by GPs in obtaining referrals for specialists, schedule inflexibility, workforce issues etc. - as opposed to an expedited entry into secondary care via presentation of severe symptoms at ED); perceived time pressure in consultations and lack of GP continuity leading to ineffective communication; GP’s lack of knowledge about interpreting symptoms and accessing the appropriate pathways; regional differences to access in services (specialist numbers, distance to medical centre); GPs’ nihilistic attitude towards lung cancer; lack of respect for and openness to other (indigenous/ ethnic minority) worldviews; lack of cultural competence and interpersonal skills	Misinterpretation or minimization of symptoms; smoking beliefs and health expectancies (many felt respiratory symptoms and generalized ill-health were the norm for smokers, or others felt that protective behaviours e.g. exercise, diet could offset health risk); individual beliefs about cancer (denial of risk, avoidance of unpleasant knowledge, shame about smoking, fears about treatment and fatalism that cancer treatment will have little benefit)	Symptom experience: lack of symptom presentation
Birt et al. (2014)^d^ [10]	M: qualitative (interviews) S: Hospital – email invitation sent to patients referred via urgent, routine and diagnostic routes	England	“white” and other	Patients (with lung cancer diagnosis and other respiratory conditions)	35 (17 lung cancer patients)	Limited access to healthcare	Misattribution of symptoms; self-management of symptoms; inability to communicate symptoms/condition; competing responsibilities	Symptom experience: co-morbidities masking respiratory changes, difficulty in recognising symptoms.
Scott, Crane, Lafontaine, Seale & Currow (2015)^a^ [31]	M: qualitative (interviews) S: lung cancer support networks	Australia	Not reported	Patients and GPs	30 (20 patients)	Increased societal awareness of lung cancer as smoking related and being the ‘fault of the individual’ (increases stigma).	Views of lung cancer as a ‘death sentence’ and severe health consequence of smoking (as portrayed in some anti-smoking messaging) meant that patients were hesitant to seek medical advice for symptoms; anticipation of stigma and blame from health professionals and general community.	
Black et al. (2015)^)c^ [27]	M: qualitative (interviews) S: EDs – member of the clinical team	England	“White British”	Patients	27 (4 lung cancer patients)	Health care professional’s appraisal leading to patients re-evaluating their symptoms inappropriately (e.g. lung cancer misdiagnosed as asthma)		
Page, Bowman, Yang & Fong (2016)^a^ [32]	M: qualitative (interviews) S: flyer, approaching people on the street, local meetings, places of employment, community centres, and snowballing	Australia	Aboriginal and Torres Strait Islander peoples	Patients, indigenous health workers and community members	67 (2 patients)		Lack of (public or private) transport to specialist health care; cost incurred in accessing care	
Wagland et al. (2016) [28]	M: mixed methods (questionnaire, clinical records review and interviews) S: GP practices – participants identified via questionnaire mailed to them	England	“White”, mixed, “Black/Black British”, “Asian/British Asian”, Chinese, other	Patients	908 (38 patient interviews)	Difficulty accessing appointments and time wasted in waiting rooms	Participants’ ‘wait and see’ attitudes towards most symptoms; guilt for symptoms perceived as self-inflicted; fear among patients for wasting GP’s time; patients not fully reporting true smoking habits or symptoms	
Caswell et al. (2017) [12]	M: qualitative (interviews) S: Hospital – participants identified through previous phase of project (not reported in this paper)	England	Not reported	Patients and carers	23 (13 patients, 10 carers)	Lack of in-depth knowledge of lung cancer signs and symptoms - GP inability to recognise symptoms and thus attributing symptoms to other conditions	Patient’s inability to recognise symptoms as pertaining to lung cancer	Symptom experience
Murray et al. (2017) [33]	M: qualitative (interviews) S: General Practice – computerized records	Australia	Not reported	Patients	20	GP communication: leading to a lack of established trust between GP and patient; GP not understanding or relating to addiction and thereby diagnosis; GP lecturing	Patient’s fear and fatalism; symptom normalisation; smoking-related guilt and stigma; past GP experiences (patients being put off by perceptions of lecturing or reprimanding to cease smoking); perception of miscommunication between patient and GP (not understanding diagnosis)	
Rankin et al. (2017)^a^ [34]	M: qualitative (interviews and focus groups) S: Hospitals – treating clinicians including pulmonologist, medical oncologist, or nurse coordinator	Australia	Not reported	Patients and GPs	30 (19 patients)	Lengthy period of time before GPs took patient concerns seriously; lengthy time intervals between diagnosis and treatment commencement; geographical location (distance) of regional health care services/GPs; lack of psychosocial support for both patient and family member(s)	Patient’s financial status	

^a^Study included multiple groups of participants including patients, barriers listed were identified by patients diagnosed with lung cancer (and family and/or carers)

^b^Study discusses intervention to reduce time to presentation with symptoms of lung cancer

^c^Study included multiple types of cancer including lung, prostate, breast and colorectal. For the purpose of this review only the barriers specific to patients diagnosed with lung cancer were included

^d^Study included patients with symptoms suggestive of lung cancer, including patients prior to a lung cancer diagnosis and patients post lung cancer diagnosis. For the purpose of this review only the barriers specific to patients post lung cancer diagnosis were included

Six studies were undertaken in the United Kingdom [10–12, 26–28], six in Australia [29–34], one in the United States [35], and one in New Zealand [36]. The ethnicities of the populations studied were European (New Zealand European, “White British”, “White”), Māori, Aboriginal and Torres Strait Island peoples, “Black/Black British”, “Asian/British Asian”, Chinese, “mixed” and other. Seven studies reported that they recruited participants from hospitals, four from community or other support groups, three from General Practices and one from a cancer register.

Five studies included multiple participant groups including patients, family and/or community members, GPs and other service providers [26, 29, 31, 32, 34]. However, as the purpose of this review was to identify barriers to early presentation and diagnosis of lung cancer specifically by patients and families or carers, only the statements made by these participants were included in our analysis. Two studies focused on multiple types of cancer including lung cancer [27, 30] – only statements by participants with a lung cancer diagnosis were included in this analysis. One study had a primary focus on the development of an intervention to reduce time to presentation with symptoms of lung cancer alongside barriers to early presentation and diagnosis [26] - the present review considered only the barriers, rather than the intervention discussed in this article. One study included patients with symptoms suggestive of lung cancer, including patients who had not yet received a lung cancer diagnosis and patients post lung cancer diagnosis [10] – this review considered only the barriers specific to patients post lung cancer diagnosis. Two studies used mixed methods (both qualitative and quantitative) for data collection [28, 30] – only qualitative data from these studies were considered for the present review. Healthcare provider and system factors as barriers to early presentation and diagnosis were identified in 13 articles, patient factors in 12 and disease factors in five.

The age and gender of participants were reported by most articles. When reported, age was provided either as an age range or mean age. Accordingly, participant age ranged from 39 to 86 years, with mean age ranging from 60 to 79 years. A good gender mix was also included in the studies reviewed.

Our findings were grouped into three categories: healthcare provider and system factors, patient factors and disease factors that serve as barriers to early presentation and diagnosis of lung cancer. These categories were based on the “contributing factors” section of Walter et al’s [7] model, as discussed previously. The following sub-sections present our results for each category.

### Healthcare provider and system factors

Healthcare provider and system factors included issues relating to delivery and healthcare policy, and barriers to access. Primarily, the quality of the relationship between GPs and patients was a recurring theme reported in many of the articles. For instance, a lack of an established relationship between patient and GP affected the quality of care provided to the patient. The quality of communication between the patient and GP resulted in a lack of established trust between patient and GP, a lengthy period of time before GPs took the patient’s concerns seriously and inadequate information provided to patients [29, 33–36]. Such barriers were also exacerbated by a lack of GP continuity [35, 36]. Specific barriers identified were, GPs’ ‘nihilism’ towards lung cancer [36], and inability to understand or relate to tobacco addiction [33]. A New Zealand study also reported that a lack of openness to other (indigenous/ ethnic minority) worldviews was a barrier to diagnosis of lung cancer [36].

Broader system factors were also identified as barriers (regardless of country level contexts), including difficulty making or accessing appointments, discontinuity of care (relating to GPs, specialists and/or other healthcare providers), long waiting times, patients getting delayed in the system or difficulty faced by GP to get referrals for specialists [26, 28, 34–36]. Patients additionally observed that limited access to health care (provider and services) was a barrier to diagnosis and care [10, 30, 34, 36].

Patients and carers also stated that GPs had inadequate knowledge of lung cancer symptoms and treatment options available. A number of studies indicated that GPs lacked knowledge about interpreting symptoms and accessing appropriate treatment pathways [12, 36]. According to Black et al. [27], patients indicated that their health care professional’s appraisal led to an inaccurate re-evaluation of self-diagnosed symptoms (e.g. symptoms of lung cancer being diagnosed as asthma).

Finally, Scott et al. [31] observed that in Australia, an increased societal awareness of lung cancer as being smoking related and being the ‘fault of the individual’, increased stigma related to the condition and smoking, thus serving as a barrier to seeking help. Moreover, according to Tod et al. [11] in the United Kingdom, media messages reinforced the fact that people should not use primary care services unless a problem was extreme.

### Patient factors

Patient factors included demographic, psychological, social and cultural factors and previous experience. A key patient related barrier recurrent in the literature was normalisation, misattribution, misinterpretation, minimization or low risk perception of symptoms relating to lung cancer [10–12, 29, 30, 33, 35, 36]. For instance, while many patients felt that respiratory symptoms and generalized ill-health were normal for smokers, others felt that protective behaviours such as exercise or diet could offset health risk. Consequently, patients engaged in self-management of symptoms rather than seeking medical advice [10, 35, 36].

Fatalistic beliefs and fear of death and/or cancer diagnosis were additionally reported as preventing patients from seeking help, often due to patients’ lack of awareness of lung cancer treatments [11, 31, 33, 35, 36]. Patients also indicated that perceived blame, stigma, guilt and shame related to smoking and diagnosis functioned as barriers [11, 28, 31, 33, 36]. Patients were put off visiting healthcare professionals by perceptions that they would be lectured or reprimanded to cease smoking [33]. ‘Stoicism’ was also reported as a barrier, particularly amongst men, where patients did not wish to complain, instead, putting on a ‘brave face’ [11, 30].

Finally, barriers related to the financial aspects of cancer care, and thus patients’ socioeconomic status, such as the high cost of health insurance or treatment and care (e.g. in the United States and Australia), lack of transport to healthcare centre (e.g. in rural Australia) and competing responsibilities (e.g. in the United Kingdom) were identified as barriers to symptom presentation and diagnosis [10, 29, 32, 34, 35].

### Disease factors

Disease factors included site, size and tumour growth rate as well as symptom presentation. Five articles reported disease factors. All of these studies indicated that symptom presentation, specifically, the wide variation in lung cancer symptoms and therefore a lack of a clear symptom profile or a lack of symptom presentation overall, made both GP diagnosis and patient awareness difficult [11, 12, 30, 36]. For example, Birth et al. (in 2014) [10] reported that the existence of co-morbidities masked many of the symptoms indicative of lung cancer (e.g. pain symptomatic of lung cancer was attributed to a kidney infection based on patient’s history of gallstone related pain, cough attributed to patient’s existing chronic respiratory symptoms or allergy).

## Discussion

This systematic literature review provided evidence that the reasons for delays in early presentation and diagnosis of lung cancer are complex and multifaceted. It is also clear that all these factors (i.e. healthcare provider and system, patient and disease) overlap. For instance, a key patient and carer perceived barrier relates to the relationship between patients and GPs. Such relationships are crucial to presentation and diagnosis of lung cancer, as they affect the level of trust between GPs and patients, patient attitudes towards their GP and vice versa, and patient perceived blame, stigma, lecturing and reprimanding by GPs [29, 33, 35, 36]. Thus, barriers relating to the relationship between patients and GPs span both healthcare provider and system factors as well as patient factors. Additionally, this review provided evidence that issues relating to access, spanning both healthcare provider and system factors and patient factors, was another key area that posed barriers to patients’ help-seeking behaviour [10, 26, 28, 32, 34, 36]. A lack of awareness of lung cancer symptoms and treatment was also identified as a significant barrier. Issues relating to a lack of awareness spanned healthcare provider and system factors, patient factors as well as disease factors, and affected patients, GPs and the general public [11, 12, 27, 31, 32, 36].

There is a clear indication in the research of the pressing need to increase lung cancer awareness, and to provide resources and knowledge regarding symptoms and treatment to patients, healthcare providers and the general public. In particular, research by Tod et al. (2008) [11], included in this review, indicates that some information campaigns relating to lung cancer were seen to contribute to fatalistic views due to a focus on death rather than treatment and/or survival. Since then, however, various awareness campaigns about the early diagnosis and/or detection of lung cancer have been trialled in New Zealand, Australia [37], Scotland [26] and Doncaster, United Kingdom [38], some of which have resulted in an increase in at-risk patients’ intentions to see a GP and request a chest X-ray (e.g. [38]). The programme implemented in Doncaster, additionally involved a brief GP education intervention for primary care practices in high lung cancer risk localities, resulting in an increase in chest X-rays and lung cancer diagnosis [38]. In many countries, clinical guidelines and optimal care pathways specific to lung cancer exist, aimed at increasing GP awareness of the disease (e.g. according to the Ministry of Health, New Zealand [39]). While such initiatives are promising, there is a need for replication, rigorous outcome evaluation [40], and to create a multi-pronged approach to raise lung cancer awareness [36]. The findings of this review, as well as that of quantitative, population level studies identifying patient perceived barriers to lung cancer diagnosis, both indicate that an inability to recognize symptoms and the stigma associated with lung cancer posed significant barriers to early diagnosis [41–44]. Thus, there is also a need to provide education to patients about the risks and symptoms of lung cancer, to dispel negative (fatalistic and stigmatising) beliefs about the disease and outcomes, and to empower at-risk patients to get checked in primary care [36]. Such an approach needs to also involve a GP training or education element, as an increase in lung cancer awareness needs to occur in patients, the general public as well as GPs and other healthcare professionals.

Delays within the system were also identified as a major barrier to presentation and diagnosis of lung cancer. For instance, delays in getting appointments, in waiting times, in getting referrals, or getting a diagnosis, the distance and access to health care providers, as well as the financial aspects of cancer care (e.g. cost of treatment, patients’ socioeconomic status) hindered access to services, and thus timely diagnosis and treatment [10, 28–30, 34–36]. Similar findings were reported by Sood et al’s (2009) [45] review of patients’ clinical records identifying barriers to diagnosis of lung cancer. Delay, irrespective of reason, can be frustrating for many patients, and when combined with difficulties accessing information and services, could increase distress [46]. It is clear that a more patient-centred and accessible approach to cancer diagnosis and care is needed.

Furthermore, many studies in our review did not report the ethnicity, or rather the ethnic variation, of their participants [10–12, 26, 29–31, 33–35]. In particular, Sharf et al. (in 2005) [35] and Tod et al. (in 2008) [11] indicated that the fact that their participant bases comprised primarily ‘white’ patients, rather than ‘black’ or minority groups, was a limitation of their research. Considering the poorer outcomes relating particularly to ethnic minority and indigenous populations diagnosed with lung cancer [47–52], the findings of this review imply that more qualitative research needs to be conducted and published with a specific focus on ethnic minority and indigenous groups. These findings also hold implications for broader arguments emphasizing the importance of culture, and of acknowledging and respecting diverse worldviews, particularly in cancer care. For example, research from Australia (not included in this review), indicates that a lack of cultural competence by GPs was a significant barrier to early presentation and diagnosis of cancer [53–56]. Such conclusions are consistent with the statements of Māori participants in Walton et al’s [36] New Zealand study, which indicated that a GP’s lack of respect for, and openness to other (indigenous/ethnic minority) worldviews posed a significant barrier to help-seeking behaviour.

Accordingly, the New Zealand Medical Council has made cultural competency training a specific core expectancy in ongoing medical education for doctors and specialty training programmes, partly to address such issues with indigenous communities [57]. In saying this, it is important to recognise that a healthcare professional’s cultural understanding of, and engagement with a patient should not be reduced to a simple set of technical skills acquired solely through cultural competency training [58]. Accordingly, the findings of this review reiterate the importance of the need for a focus on building relationships between patient and GP. As such, the Australian studies report that many Aboriginal Australians hold differing health beliefs of cancer causation [53–56]. For instance, this can include a belief that cancer is contagious, or simply the lack of a word for ‘cancer’, resulting in the diagnosis and its implications not being understood by many of these groups [55]. Accordingly, these researchers indicate that there needs to be an acknowledgement of such differing worldviews by the broader healthcare system, and that GPs need to also be aware of the significance of traditional healing methods germane to each of these communities [54, 56]. While we acknowledge that not all indigenous communities are the same, there is need for health professionals to have knowledge of, and/or experience in, not only medicine, but also the communities they serve, which goes beyond a simple set of skills acquired through cultural competency training. Overall, more attention needs to be paid to identifying and addressing barriers to early presentation and diagnosis of lung cancer among indigenous communities.

A number of studies in this review also indicated that patients, or potential participants of their research, died prior to the commencement of interviews as a result of lung cancer (e.g. [12, 29, 30]). This was listed as a limitation of such studies. The fact that lung cancer patients passed away within the short timeframe of a recruitment process reinforces the importance and urgency of identifying and addressing the barriers to early presentation and diagnosis of lung cancer.

The strengths of this review were that it assessed 14 high quality studies from respected journals, bringing together statements from a total of 240 patients from five countries and diverse populations. A limitation of this review was that it only examined studies published in English. However, the consistency of results identified in these studies provides some reassurance as to their validity. Studies reviewed were also from a limited number of countries. Research from countries that are not considered First World nations may have contributed significantly to our findings. Moreover, we documented only the perceived barriers identified by patients and carers. Patients and carers are the most valid source for identifying barriers to early presentation and diagnosis of lung cancer. However, understanding GP views alongside population level data may be necessary in order to introduce effective interventions.

## Conclusion

Early presentation and detection of symptoms relating to lung cancer is critical to improving survival. Delays in early presentation and diagnosis of lung cancer might be avoided if various barriers relating to healthcare provider and system factors as well as patient and disease factors are addressed. This paper provides a complete, exhaustive summary of current patient-centred evidence identifying the existent barriers to early diagnosis of lung cancer, by bringing together and reviewing 14 qualitative studies from various countries. According to the findings of our review, a good starting point to addressing patient and carer perceived barriers, is to focus on the three key areas of relationship building between GP and patient, improving patient access to services and care, and increasing awareness of lung cancer symptoms and treatment, particularly among disadvantaged communities.

## Abbreviations

CASP checklist: Critical Appraisal Skills Programme checklist
GP: General Practitioner
LDCT: Low Dose Computerised Tomography
PRISMA guidelines: Preferred Reporting Items for Systematic Reviews and Meta-Analyses guidelines
